# Characteristics of hospitalized elderly patients with CKD: a comparison between elderly and non-elderly CKD based on a multicenter cross-sectional study

**DOI:** 10.1007/s11255-023-03675-1

**Published:** 2023-07-14

**Authors:** Shuang Liang, Yong Wang, Wen-Ling Wang, Xin-Ru Guo, Chun Zhang, Chen Yang, Che Wang, Guang-Yan Cai, Xiang-Mei Chen

**Affiliations:** 1 Department of Nephrology, First Medical Center of Chinese PLA General Hospital, Nephrology Institute of the Chinese People’s Liberation Army, State Key Laboratory of Kidney Diseases, National Clinical Research Center for Kidney Diseases, Beijing Key Laboratory of Kidney Disease Research, Beijing, China; 2 Department of General Internal Medicine, The Fifth Medical Center of Chinese, PLA General Hospital, Beijing, China

**Keywords:** Chronic kidney disease, Elderly, Etiology, Comorbidities, In-hospital outcomes

## Abstract

**Purpose:**

We undertook a multicenter epidemiological survey among hospitalized patients with chronic kidney disease (CKD), aiming to reveal the characteristics of elderly CKD by comparing it with non-elderly CKD.

**Methods:**

Medical records were obtained from 18 military hospitals across China from 1 January 2009 to 31 December 2011. The characteristics of chronic kidney disease in the elderly were analyzed through comparing with those in younger patients with chronic kidney disease.

**Results:**

A total of 380,461 hospitalized patients were included in the database, with 25,826 (6.8%) diagnosed with CKD. Unlike non-elderly, the top-three causes of chronic kidney disease among elderly patients were diabetic nephropathy (24.1%), hypertension-related renal impairment (20.9%), and primary glomerular disease (11.1%). 71.6% of the elderly patients with CKD had more than one comorbidities and the number of morbidities increased with age. In-hospital mortality of the elderly was significantly higher than those of younger patients (3.3% vs. 1.0%). Multiple logistic regression analysis showed that age, CKD 5 stage, acidosis, cardiovascular and cerebrovascular diseases, infection disease, neoplasm, and dementia were independent risk factors for death from CKD in the elderly. The median length of stay (LOS) was similar between elderly and younger CKD patients. The median cost was higher for elderly CKD patients than for younger CKD patients. Among elderly individuals with CKD, LOS, and hospitalization costs also increased with an increase in the number of coexisting diseases.

**Conclusions:**

Diabetic nephropathy,  and hypertension-related kidney injury were the leading causes of chronic kidney disease in elderly patients, which is different from the non-elderly. Elderly patients with chronic kidney disease were more likely to have a higher burden of comorbidities, which were associated with worse in-hospital outcomes.

**Supplementary Information:**

The online version contains supplementary material available at 10.1007/s11255-023-03675-1.

## Introduction

It is expected that the proportion of the world’s population over 60 years of age will increase from nearly 11% currently to 22% by 2050 [1]. In parallel with this situation, there has been a steady increase in the prevalence of age-associated chronic disease and disability [2, 3], including chronic kidney disease (CKD). CKD is a significant public-health problem because of the poor outcomes of patients and significant financial burden upon families and society [4, 5]. CKD prevalence increases with age, and being over 60 years of age is considered an independent risk factor for CKD [6, 7]. A survey in China reported the CKD prevalence among those aged 18–39, 60–60, and > 70 years to be 7.4%, 18%, and 24.2%, respectively [8]. Similarly, data from the National Health and Nutrition Examination Survey suggest that CKD prevalence is 38% in participants aged > 65 years compared with 13% in the overall US population [9]. In addition, it has been reported that the median age of new dialysis patients is 65 years, and that the number of people aged > 75 years requiring dialysis is increasing rapidly [10].

Control of CKD among elderly people is challenging because of its heterogeneity, the increased risk of complications and comorbidities, frailty, social isolation, and poor functional status. Therefore, therapies based on evidence generated in younger CKD groups may not be applicable to older patients. However, there is a paucity of multicenter studies on CKD among elderly people in China. We aimed to conduct a multicenter epidemiological survey and provide data on characteristics [including demographics, etiologies, comorbidities, in-hospital mortality, length of stay (LOS), and cost] among aging patients with CKD by comparison with younger CKD, which was important for better detection and control of CKD among elderly people.

## Patients and methods

### Ethical approval of the study protocol

The study protocol was approved by the Medical Ethics Committee of the Chinese PLA General Hospital (Approval No. of Ethics Committee S2022-012-02, Beijing, China). The requirement for written informed consent was waived by this institution due to the retrospective nature of the study. Decisions letter of Ethics Committee has been attached, which cover patient data confidentiality and compliance with the Declaration of Helsinki.

### Study design

This was a cross-sectional analysis of a national dataset held by the Chinese PLA General Hospital. The dataset consisted of copies of clinical data for all registered patients from 18 hospitals. All participating clinical centers were tertiary hospitals, located in 15 provinces, in a six-region partition of China (north, northeast, east, south central, southwest, and northwest). The detailed distribution of the 18 hospitals is given in Online Supplementary Information (Supplemental Table 1). Participants aged ≥ 18 years who received a diagnosis of CKD from 1 January 2009 to 31 December 2011 were enrolled.

### Data collection

Patient-level data were extracted from the uniform “front page” of the hospital medical record. The front page has legal validity and is completed by physicians who have the most accurate and comprehensive understanding of the patient’s medical condition. Then, the diagnoses were coded according to the International Classification of Diseases, 10th edition, clinical modification 2008 (ICD-10) coding system by certified professional medical coders at each hospital. For each patient, the following variables were documented: (i) demographics [identification (ID) number, age, gender, ethnicity, number of hospitalizations, patient residence, location of the hospital, admission/discharge department, admission/discharge date, and length of stay (LOS)]; (ii) clinical diagnosis (ICD-10), procedures, and surgical procedures; (iii) all-cause in-hospital death, and total cost. Urban and rural divisions of patient residence were divided according to the standards issued by the National Bureau of Statistics (http://www.stats.gov.cn/). The ID number of each patient combined with the number of hospitalizations is unique and serves as a screening indicator. For people hospitalized multiple times during the study period, information about the first hospitalization was collected.

### Definitions

#### Identification of CKD patients

Diagnosis codes from ICD-10 were used to identify patients with CKD in 18 hospitals according to the database. The etiologies and corresponding disease codes are listed in Supplemental Table 2.

#### Definition of “elderly”

Adults over 65 years of age were defined as “elderly”. People aged 18–64 years were classified as “non-elderly”.

#### Definition of “hypertension-related kidney injury”

In the database, a certain percentage of patients were diagnosed with “chronic renal failure”, without other etiology. Among them, those diagnosed with “primary hypertension” were classified as “CKD + HT of unknown cause”, and the others were classified as “CKD of unknown cause”. “Hypertension-related kidney injury” was defined as hypertensive nephropathy, and CKD + HT of unknown cause.

#### Definitions of “comorbidities”

A standard approach for assessment of comorbidities is lacking. Hence, selection and definition of the morbidities to include are inevitably, to some degree, subjective and dependent upon the data available. According to the Charlson Comorbidity Index [11] and the 40 common coexisting diseases listed in some studies [12], we selected 12 comorbidities defined by ICD-10 (Supplemental Table 3). In this part, hypertension and diabetes were comorbidities, not etiologies.

### Statistical analyses

Continuous variables are expressed as the mean ± SD or median (interquartile range). Student’s *t* test or one-way ANOVA was used for continuous variables. Categorical variables are expressed as n (%) and were compared by the *χ*^2^ test. Logistic regression analysis was used to explore the risk factors for death from CKD in the elderly. *P* < 0.05 (two-tailed) was considered significant. Excel™ (Microsoft, Redmond, WA, USA) charts were used to establish a case-file database, and analyses were processed with SPSS 22.0 (IBM, Armonk, NY, USA).

## Results

### Detection of CKD in the elderly

A total of 380,461 hospitalized patients were included in the database, with the average age of 60.4 ± 13.8 years. The vast majority of the study population was of Han ethnicity (368,159, 96.8%). Patients with CKD constituted 6.8% of all inpatients. The percentage with CKD was relatively high in those aged 18–44 years, compared with those aged 45–64 years, and ≥ 65 years. The overall distribution of gender was similar in the database, and was consistent across age groups (Supplemental Table 4). Among elderly patients with CKD, 65.3% were admitted and discharged from a renal department, followed by intensive-care unit (ICU) (4.4%), infection department (3.9%), geriatrics department (3.2%), and surgery department (2.6%). With regard to the geographic region of patient residency, the highest percentages of elderly CKD patients were in Southwest (5.5%) and Northeast (5.1%).

### Etiology of elderly patients with CKD

Except for chronic renal failure of unknown cause, primary glomerulonephritis remained the most prevalent cause of CKD in younger patients. However, the top-three causes of CKD among elderly patients were diabetic nephropathy (24.1%), hypertension-related kidney injury (20.9%), and primary glomerulonephritis (11.1%). The prevalence of diabetic kidney disease, hypertension-related kidney injury, neoplastic diseases, obstructive nephropathy, and chronic tubulointerstitial nephritis among elderly patients was significantly higher than that in non-elderly patients. The prevalence of primary glomerulonephritis, kidney disease due to autoimmune diseases, congenital and hereditary nephropathy, and infection-related nephritis was significantly lower in elderly patients than that in non-elderly patients (Table 1). Out of 5741 participants with primary glomerulonephritis, 1465 (25.5%) had available data on the pathological diagnosis. The most common pathological types of primary glomerular disease in the elderly were membranous nephropathy (MN) (54.6%), IgA nephropathy (IgAN) (31.1%), and mesangial proliferative glomerulonephritis (13.6%). The most common pathological types of primary glomerular disease in the non-elderly were IgAN (48.0%), mesangial proliferative glomerulonephritis (18.0%), and MN (15.2%).

**Table 1:** Etiology of chronic kidney disease

Etiology of CKD	Overall		Elderly		Non-elderly		*P*
	No. of patients	%	No. of patients	%	No. of patients	%	
CKD of unknown cause	7689	29.8	1958	28.1	5731	30.4	< 0.001
Primary glomerulonephritis	5741	22.2	775	11.1	4966	26.3	< 0.001
Diabetic nephropathy	4764	18.5	1675	24.1	3089	16.4	< 0.001
Type-1 diabetes mellitus	71	0.3	4	0.1	67	0.4	< 0.001
Type-2 diabetes mellitus	3530	13.7	1216	17.5	2314	12.3	< 0.001
Other specified diabetes mellitus	1	0	0	0	1	0.0	–
Unspecified diabetes mellitus	1162	4.5	455	6.5	707	3.8	< 0.001
Hypertension-related kidney injury	3721	14.4	1456	20.9	2265	12.0	< 0.001
CKD + HT of unknown cause	2463	9.5	953	13.7	1510	8.0	< 0.001
Hypertensive nephropathy	1258	4.9	503	7.2	755	4.0	< 0.001
Neoplastic diseases	1110	4.3	472	6.8	638	3.4	< 0.001
Malignant neoplasm of kidney	942	3.7	407	5.8	535	2.8	< 0.001
Myeloma nephropathy	92	0.4	28	0.4	64	0.3	0.454
Amyloid nephropathy	14	0.1	3	0.0	11	0.1	–
Kidney damage due to light chain deposition disease	1	0	0	0	0	0	
Renal neoplasms of uncertain or unknown behavior	61	0.2	34	0.5	27	0.1	< 0.001
Obstructive nephropathy	842	3.3	270	3.9	572	3.0	0.001
Tubulointerstitial nephritis	704	2.7	258	3.7	466	2.4	< 0.001
Autoimmune	642	2.5	69	1.0	573	3.0	< 0.001
Congenital and hereditary nephropathy	628	2.4	83	1.2	545	2.9	< 0.001
Infection-related nephritis	210	0.8	10	0.1	200	1.1	< 0.001
Other	1078	4.2	244	3.5	834	4.4	0.001
CKD due to other certain causes	676	2.6	191	2.7	485	2.6	0.447
CKD of transplanted kidney	283	1.1	11	0.2	272	1.4	< 0.001
Gout and hyperuricemia nephropathy	119	0.5	42	0.6	77	0.4	0.04

*CKD* chronic kidney disease

**Fig. 1:**
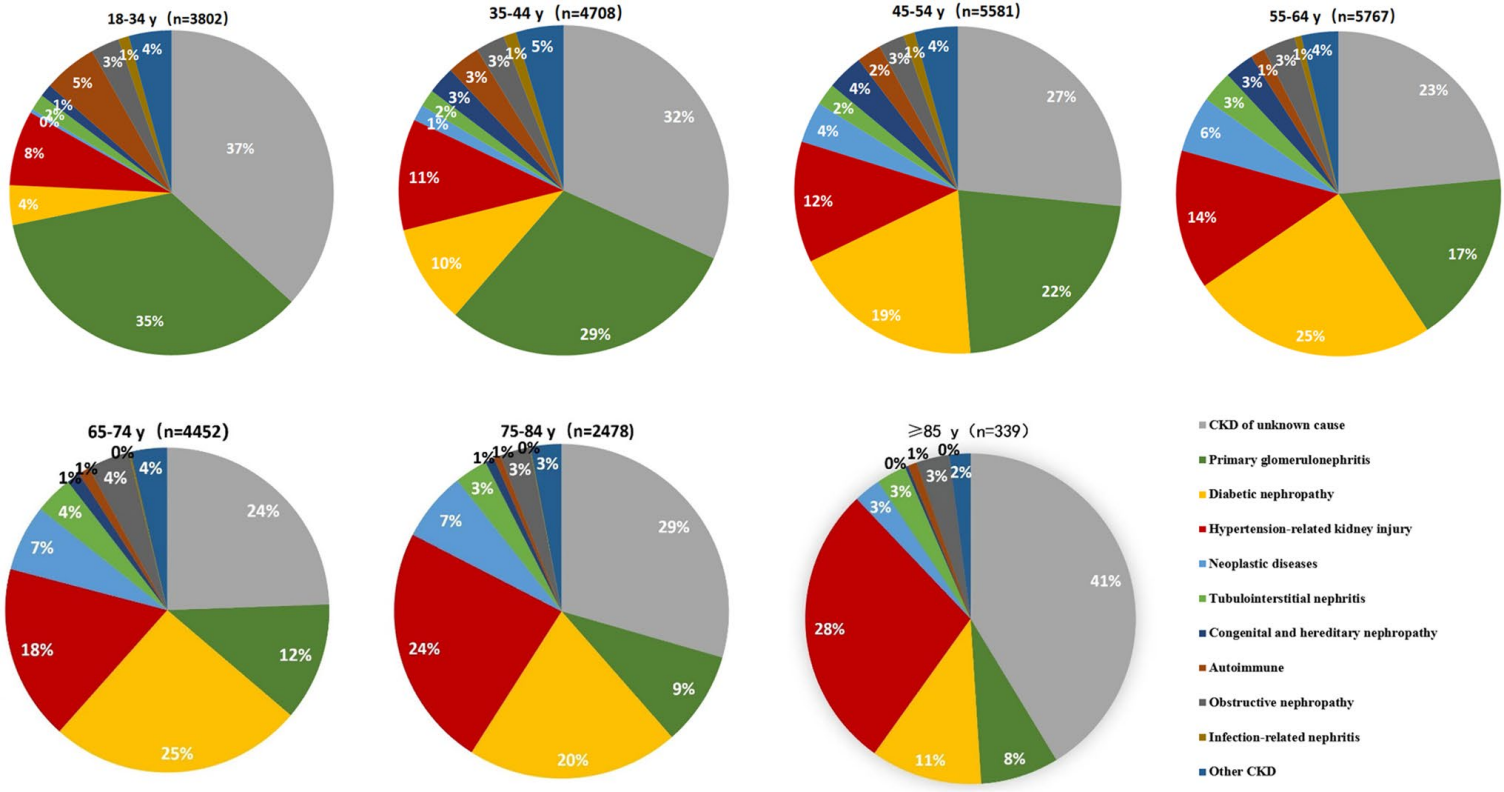
The etiologies of CKD in different age groups

The etiologies of CKD in different age groups are shown in Fig. 1. The prevalence of primary glomerulonephritis was highest in those aged 18–34 years (35%), and decreased gradually with age. The prevalence of diabetic nephropathy increased progressively from < 10% in those aged < 45 years to nearly 20% in people aged 45–54 years, reached a peak of 25% in people aged 55–75 years, and declined to 11% in the 85 + years age group. Middle-aged and elderly people, especially those 55–85 years of age, had the highest prevalence of tumor-related kidney injury (6–7%).

### Comorbidities of elderly patients with CKD

Elderly CKD patients were much more likely than younger CKD patients to have most of the 12 comorbidities. The prevalence of systemic connective-tissue disorders was lower in elderly patients with CKD than in younger patients. There was no significant difference in the prevalence of infectious diseases, liver diseases, or acute kidney injury (AKI). Among elderly patients with CKD, the three most prevalent comorbidities were hypertension (33.8%), diabetes (29.7%), and cardiovascular and cerebrovascular diseases (21.7%) (Table 2).

**Table 2:** Comorbidities of patients with chronic kidney disease

Comorbidities of CKD	Overall (*n* = 25,826)		Elderly (*n* = 6966)		Non-elderly (*n* = 18,860)		*P*
	*n*	%	*n*	%	*n*	%	
Hypertension	8104	31.4	2352	33.8	5752	30.5	< 0.001
Diabetes mellitus	5856	22.7	2067	29.7	3789	20.1	< 0.001
Cardiovascular and cerebrovascular diseases	3141	12.2	1512	21.7	1629	8.6	< 0.001
Infectious diseases	1186	4.6	311	4.5	875	4.5	0.551
Neoplasms	877	3.4	389	5.6	488	2.6	< 0.001
Liver diseases	591	2.3	145	2.1	446	2.4	0.177
Systemic connective-tissue disorders	577	2.2	62	0.9	515	2.7	< 0.001
Diseases of the digestive system	409	1.6	159	2.3	250	1.3	< 0.001
Diseases of the respiratory system	286	1.1	194	2.8	92	0.5	< 0.001
AKI	206	0.8	58	0.8	148	0.8	0.701
PVD	190	0.7	74	1.1	116	0.6	< 0.001
Dementia	8	0.0	8	0.1	0	0	–

*CKD* chronic kidney disease, *AKI* acute kidney injury, *PVD* peripheral vascular disease

Elderly CKD patients tended to have multiple comorbidities compared with non-elderly patients. Only 28.4% of elderly CKD patients had a single renal diagnosis, and 71.6% had one or more comorbidities. The number of comorbidities, and the prevalence of multiple comorbidities, increased with age (Table 3).

**Table 3:** Comorbidities in different age groups

No. of comorbidities	Average age (years)	Overall (*n* = 25,826)		Elderly (*n* = 6966)		Non-elderly (*n* = 18,860)		*P*
		*n*	%	*n*	%	*n*	%	
0	49.3	9968	38.6	1979	28.4	7989	42.4	< 0.001
1	54.8	11,064	42.8	3048	43.7	8016	42.5	0.071
2	59.2	4064	15.7	1572	22.6	2492	13.2	< 0.001
3	63.3	688	2.7	341	4.9	347	1.8	< 0.001
4 +	66.8	41	0.2	26	0.4	15	0.1	< 0.001

### In-hospital outcomes of elderly patients with CKD

#### Mortality

**Fig. 2:**
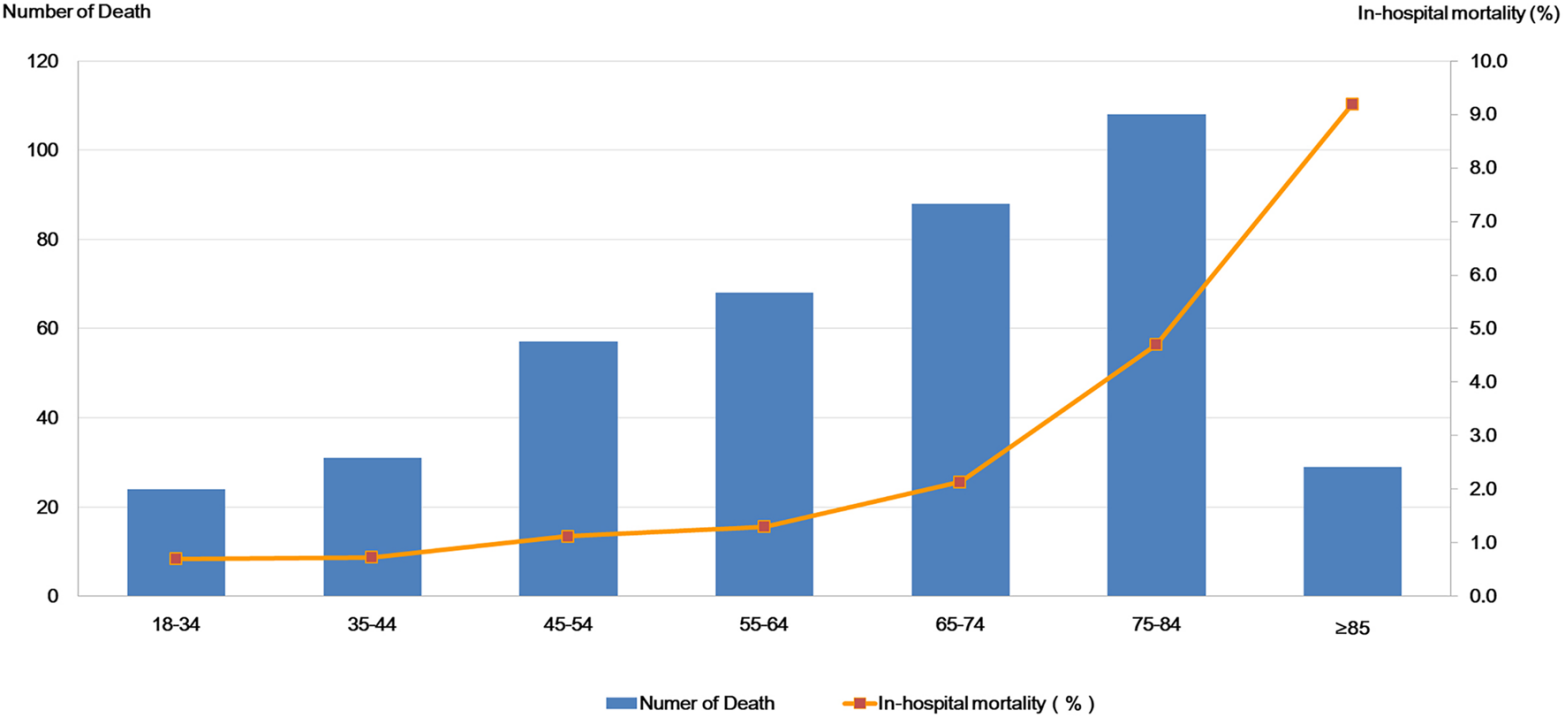
In-hospital mortality by age group

Among the 25,826 CKD patients, those with missing data on treatment results were excluded (*n* = 1030), leaving 24,796 patients with recorded treatment results. In-hospital mortality of elderly CKD patients was higher than that of younger CKD patients (3.3% vs. 1.0%, *p* < 0.001). In-hospital mortality by age group increased from 0.7% in those aged 18–35 years to 9.2% in patients aged > 85 years (Fig. 2).

#### Risk factors for death from CKD in the elderly

We analyzed the characteristics of 6734 older CKD patients by comparing those who died with those who survived (Table 4). The age, the cost, and the portion of acidosis and hyperkalemia were significantly higher in the patients who died. The prevalence of comorbidities, including hypertension, diabetes, cardiovascular and cerebrovascular diseases, infection disease, neoplasm, and dementia, were higher in patients who died than whose who survived.

**Table 4:** Comparison of elderly patients with CKD who died and who survived

	Elderly CKD (*n* = 6734)	Patients who died (*n* = 225)	Patients who survived (*n* = 6509)	*P* value
Age	73.37 ± 6.01	76.72 ± 6.85	73.25 ± 5.95	< 0.001
Gender (male, %)	3823 (56.8%)	123 (54.7%)	3700 (56.8%)	0.517
Medical insurance (yes, %)	4672 (72.3%)	170 (76.6%)	4502 (72.1%)	0.146
CKD stage				
Stage 2	130 (1.9%)	0	130 (2.0%)	–
Stage 3	734 (10.9%)	10 (4.4%)	724 (11.1%)	0.002
Stage 4	226 (3.4%)	6 (2.7%)	220 (3.4%)	0.559
Stage 5	1331 (19.8%)	81 (36.0%)	1251 (19.2%)	< 0.001
Complications of CKD				
Anemia	514 (7.6%)	13 (5.8%)	501 (7.7)	0.286
Acidosis	88 (1.3%)	13 (5.8%)	75 (1.2)	< 0.001
Hypoproteinemia	32 (0.5%)	2 (0.9%)	30 (0.5)	0.290
Hyperparathyroidism	19 (0.3%)	0	19 (0.3)	–
Hyperkalemia	97 (1.4%)	8 (3.6%)	89 (1.4)	0.015
Uremic encephalopathy	19 (0.3%)	2 (0.9%)	17 (0.3)	0.131
Comorbidity				
Hypertension	2279 (33.8%)	42 (18.7%)	2237 (36.9%)	< 0.001
Diabetes	2038 (30.3%)	46 (20.4%)	1992 (30.6%)	0.001
Cardiovascular and cerebrovascular diseases	1467 (21.8%)	77 (34.2%)	1390 (21.4%)	< 0.001
Infection disease	302 (4.5%)	19 (8.4%)	283 (4.4%)	0.004
Neoplasm	339 (5.0%)	29 (12.9%)	310 (4.7%)	< 0.001
Liver disease	142 (2.1%)	9 (4.0%)	133 (2.0%)	0.055
Systemic connective-tissue disorders	62 (0.9%)	2 (0.9%)	60 (0.9%)	0.959
Diseases of the digestive system	155 (2.3%)	5 (2.2%)	150 (2.3%)	0.936
Diseases of the respiratory system	190 (2.8%)	6 (2.7%)	184 (2.8%)	0.887
AKI	57 (0.9%)	2 (0.9%)	55 (0.8%)	0.716
PVD	70 (1.0%)	2 (0.9%)	68 (1.0%)	1.000
Dementia	8 (0.1%)	2 (0.9%)	6 (0.1%)	0.027
LOS	13 (8–19)	12 (4–23)	13 (8–18)	0.053
Cost	10.3 (6.2–18.9)	22.4 (9.0–46.2)	10.2 (6.2–18.4)	< 0.001

*CKD* chronic kidney disease, *AKI* acute kidney injury, *PVD* peripheral vascular disease, *LOS* length of stay

We first screened out factors that might be associated with death from CKD in the elderly using univariable logistic regression analysis, and then identified the independent mortality risk factors using multivariable logistic regression analysis. Age, CKD 5 stage, acidosis, cardiovascular and cerebrovascular diseases, infection disease, neoplasm, and dementia were independent risk factors for death from CKD in the elderly (Table 5).

**Table 5:** Risk factors for death from CKD in the elderly

Variable	*β* value	OR	95% CI	*P* value
Age	0.084	1.088	1.065–1.110	< 0.001
CKD 5 stage	1.030	2.800	2.073–3.783	< 0.001
Acidosis	1.617	5.039	2.637–9.630	< 0.001
Cardiovascular and cerebrovascular diseases	0.837	2.309	1.711–3.116	< 0.001
Infection disease	0.656	1.928	1.160–3.203	0.011
Neoplasm	1.322	3.753	2.432–5.789	< 0.001
Dementia	2.124	8.361	1.621–43.114	0.011

*CKD* chronic kidney disease

#### LOS and cost

The median LOS was similar between elderly and younger CKD patients (13 (range 8–18) vs. 12 (8–19) days, *p* = 0.974). The median cost was higher for elderly CKD patients than for younger CKD patients (10.2 (6.2–18.9) vs. 10.0 (5.7–18.8) thousand yuan, *p* = 0.009). Among elderly individuals with CKD, the mean LOS was longest among patients had CKD and coexisting dementia (16 (7.5–34.5) days); the average hospitalization cost was highest when elderly CKD combined with tumors (17.58 (8.25–35.27) thousand yuan). LOS and hospitalization costs also increased with an increase in the number of coexisting diseases. If > 3 comorbidities were present, then the average LOS was the longest (14 (range 11–21) days), and the hospitalization cost was the highest (15.26 (range 10.72–28.12) thousand yuan).

## Discussion

We observed that patients with CKD constituted 6.8% of all hospitalized patients. The percentage of elderly hospitalized patients with CKD was 4.5%, lower than that for younger patients with CKD. This observation could have been attributable to the following reasons. First, the symptoms of estimated glomerular filtration rate (eGFR) decrease (e.g., fatigue and gastrointestinal discomfort) are often non-specific and insidious, and tend to be ignored by elderly people, which delays nephrology referrals and medical treatment. A cross-sectional study conducted in a middle-aged and elderly Chinese population showed that among patients with CKD, only 8.7% were aware of the diagnosis and only 4.9% of patients received treatment. Moreover, the level of awareness and proportion of treated patients decreased with increasing age. Apart from age, there was no significant difference in characteristics between patients aware of their diagnosis and those unaware [13]. Low awareness of CKD may contribute to the low percentage of hospitalization. Secondly, except for actual CKD prevalence and hospitalization prevalence, percentages of hospitalized CKD also based on sensitivity of CKD diagnoses. In our study, CKD was identified according to the “front page” instead of laboratory tests (e.g., eGFR and proteinuria). The low sensitivity of diagnosis codes to identify CKD has been reported in some studies, which may reflect the low awareness of CKD among physicians or coding practices [14–17]. Such data serves as a reminder that CKD management in aging populations in China is far from satisfactory, and that screening and health education are imperative, both in the elderly and medical staff.

Glomerulonephritis is used to be more prevalent in less-developed countries such as China [18]. However, in recent decades, with economic growth, as well as the change of diet patterns and lifestyles, the prevalence of metabolic diseases (e.g., obesity, diabetes, and hypertension) has increased rapidly in China [19]. We hypothesized that the increase in prevalence of diabetes and hypertension might affect the etiology spectrum of elderly patients with CKD in China. One study involving 173 CKD patients from 1990 to 1991 and 956 patients from 2009 to 2010 showed that primary glomerulonephritis remained the most prevalent cause of CKD patients age ≤ 60, while in elderly patients, the prevalence of diabetes and hypertension increased and became the leading cause of CKD in 2009–2010 [20], but the study was from a single center involving a small sample. Our study, based on a database from 18 hospitals involving 25,826 hospitalized CKD patients, indicated that diabetes and hypertension were the most common causes of CKD in elderly patients, which was confirming the prior findings. This finding suggested that aging population-based surveillance and control of diabetes and hypertension has become an important component of addressing CKD. It is suggested that albuminuria screening should be done immediately after the diagnosis of type-2 diabetes and should be undertaken from the fifth year after the diagnosis of type-1 diabetes, and annually in both types [21, 22]. In addition, differentiating normal renal degeneration caused by aging and nephropathy in a patient with diabetes should be done. There may be a need to adjust current formulas for the eGFR in patients who are both older and suffering from diabetes [23, 24]. Inhibitors of the sodium-glucose cotransporter 2 (SGLT2), a new class of antihyperglycemic drugs, has been showed to not only lower the level of glucose, but also improve renal and cardiovascular outcomes in patients with CKD [24, 25]. Hypertension is both a cause and effect of CKD [26]. Control and treatment of hypertension in elderly participants not only reduce the risk of cardiovascular events, but also slow down progression of CKD [27]. 24-Hour ambulatory blood pressure monitoring (ABPM) provides a more accurate assessment of diurnal variation in blood pressure and is a better predictor of CVD events in those with CKD than clinic readings [28]. The optimal blood pressure target in aging population with CKD remains controversial [29–31]. It is widely demonstrated that inhibition of the renin-angiotensin–aldosterone system (RAAS), like Ramipril, has been widely used to reduce proteinuria and to treat hypertension, one of the main causes of renal failure in the elderly patients [32].

One of the greatest challenges in geriatric medicine is the great burden of chronic comorbidities. Having more than one comorbidity becomes progressively more common with age [33, 34] and is associated with high mortality [35], reduced functional status [36, 37], and increased use of healthcare facilities [34, 38]. Our study involving a relatively large sample supported that elderly patients with CKD carried a higher prevalence of comorbidities than younger individuals, and the number of comorbidities increased with age. Among elderly individuals with CKD, those with more comorbidities had a longer LOS and had higher hospitalization costs. Comorbidities and their associated adverse outcomes often complicate the condition and risk modification of older adults with CKD [39–41]. Younger patients with CKD may progress to end-stage renal disease, but elderly patients are more likely to have global health outcomes (e.g., death) than suffer a decline in kidney function [42]. We identified that except for age, renal function (e.g., CKD 5 stage) and CKD-related complication (e.g., acidosis), comorbidities including cardiovascular and cerebrovascular diseases, infection disease, neoplasm, and dementia were independent risk factors for death from CKD in the elderly. These data suggest that multiple comorbidities in an aging population with CKD are important and should be taken into full consideration. First, preferences of patients should be incorporated into medical decision-making. Older CKD adults and their families or individuals representing patients should be adequately informed about the expected benefits and harms of therapies. Then, they can prioritize their preferences based on their personal and cultural contexts regarding health and healthcare [43]. Second, except for disease-specific endpoints (e.g., progression to end-stage renal disease), outcome measures, such as life expectancy, functional status, wellbeing, and other health-related quality-of-life assessments, should also be evaluated [44–47]. One example is the use of glucocorticoids in some types of glomerulonephritis. Benefits include a lower risk of renal-function decline and proteinuria, but application of glucocorticoids also increases the risk of infection, femoral-head necrosis, and gastrointestinal hemorrhage. Aging, along with the presence of other common risk factors (e.g., chronic respiratory disease, osteoporosis, and previous gastrointestinal bleeding) can dramatically amplify those risks, which should be considered and the risks and benefits should be weighed when making management decisions. Third, older adults with comorbidities are at greater risk of potential harms associated with polypharmacy and invasive procedures. Hence, clinicians should pursue more flexible approaches (e.g., reduction of medication number, identifying interventions that should not be initiated, regular monitor of the efficacy, and safety of medications).

The main strength of this study is that it was a multicenter survey of hospitalized aging population with CKD, with an ethnically diverse, and relatively large sample. Our study had some limitations. First, there were some uncertainties in the identification, etiology of CKD, and morbidities, because the definition was partly subjective and dependent on the data available from the “front page” of the medical record. Second, this was a retrospective cohort study with a cross-sectional design, and hence, causal associations cannot be inferred with certainty. More prospective studies are needed. We have conducted a national-wide prospective cohort study of elderly patients with CKD (C-OPTION), where comprehensive geriatric assessment were assessed, aimed to better understand the characteristics of this population [48].

### Supplementary Information

Below is the link to the electronic supplementary material.

Supplementary file1 (DOCX 35 KB)

## Data Availability

Data available on reanonable request from the authors.
