# Supplementary material for: Characteristics of hospitalized elderly patients with CKD: a comparison between elderly and non-elderly CKD based on a multicenter cross-sectional study
Source: Int Urol Nephrol. 2023 Jul 14;56(2):625–33. doi: 10.1007/s11255-023-03675-1 (PMC10808144; doi:10.1007/s11255-023-03675-1)
Supplement: Supplementary file 1 — Supplementary file1 (DOCX 35 KB) [file 11255_2023_3675_MOESM1_ESM.docx]

Supplemental Table 1.The distribution of the 18 hospitals and the number of hospitalized patients

| Region | No. of hospitals | No. of hospitalized patients（n,%） |
| --- | --- | --- |
| Southwest | 2 | 65720（17.3） |
| Chongqing | 1 | 60509（15.9） |
| Yunnan | 1 | 5211（1.4） |
| East | 5 | 59618（15.7） |
| Shandong | 3 | 39021（10.3） |
| Fujian | 1 | 13812（3.6） |
| Jiangxi | 1 | 6785（1.8） |
| North | 4 | 109201（28.7） |
| Beijing | 2 | 50735（13.3） |
| Tianjin | 1 | 25844（6.8） |
| Hebei | 1 | 32622（8.6） |
| Northwest | 3 | 97123（15.5） |
| Shanxi | 1 | 47685（12.5） |
| Gansu | 1 | 28461（7.5） |
| Xinjiang | 1 | 20977（5.5） |
| Northeast | 2 | 23878（6.3） |
| Liaoning | 1 | 17588（4.6） |
| Heilongjiang Long Jiang | 1 | 6290（1.6） |
| South central | 2 | 24921（6.6） |
| Guangdong Dong | 1 | 6144（1.6） |
| Henan | 1 | 18777（4.9） |

Supplemental Table 2. Coding of various etiologies of chronic kidney disease

| Etiology of CKD | ICD-10 code |  |
| --- | --- | --- |
| Other CKD of unknown cause | N18 | Chronic renal failure (excluding CKD combined  with other than N19) |
|  | N19 | Unspecified renal failure (excluding CKD combined with other than N18 and not combined with AKI) |
| Diabetes mellitus | E10.2 | Insulin-dependent diabetes mellitus with renal complications |
|  | E10.6, E10.7, E10.8 | Kidney injury in “Insulin-dependent diabetes mellitus with other specified/multiple/unspecified complications” |
|  | E11.2 | Non-insulin-dependent diabetes mellitus with renal complications |
|  | E11.6–E11.8 | Kidney injury in “Non-insulin-dependent diabetes mellitus with other specified/multiple/unspecified complications” |
|  | E12.2 | Malnutrition-related diabetes mellitus with renal complications |
|  | E13.2 | Other specified diabetes mellitus with renal complications |
|  | E13.7, E13.8 | Kidney injury in “Other specified diabetes mellitus with multiple/unspecified complications” |
|  | E14.2 | Unspecified diabetes mellitus with renal complications |
|  | E14.6–E14.8 | Kidney injury in “Unspecified diabetes mellitus with other specified/multiple/unspecified complications” |
| Hypertensive diseases | I12.0 | Hypertensive renal disease with renal failure |
|  | I12.9 | Hypertensive renal disease without renal failure |
|  | I13.0 | Hypertensive heart and renal disease with (congestive) heart failure |
|  | I13.1 | Hypertensive heart and renal disease with renal failure |
|  | I13.2 | Hypertensive heart and renal disease with both (congestive) heart failure and renal failure |
|  | I13.9 | Hypertensive heart and renal disease, unspecified |
| CKD + Hypertension | N18 + I10 | CKD + Essential (primary) hypertension (excluding CKD combined with other than N19) |
|  | N19 + I10 | CKD + Essential (primary) hypertension (excluding CKD combined with other than N18) |
| Glomerular diseases | N02 | Recurrent and persistent hematuria |
|  | N03 | Chronic nephritic syndrome |
|  | N04 | Nephrotic syndrome |
|  | N05 | Unspecified nephritic syndrome |
|  | N06 | Isolated proteinuria with specified morphological lesion |
|  | N39.1 | Persistent proteinuria, unspecified |
| Renal tubulointerstitial diseases | N11 | Chronic tubulointerstitial nephritis |
|  | N12 | Chronic tubulointerstitial nephritis in “Tubulointerstitial nephritis, not specified as acute or chronic” |
|  | N14 | Drug- and heavy metal-induced tubulointerstitial and tubular conditions |
|  | N15 | Other renal tubulointerstitial diseases |
|  | N16 | Renal tubulointerstitial disorders in diseases classified elsewhere (excluding N16.4) |
|  | E74.8 | Other specified disorders of carbohydrate metabolism |
|  | E72.0 | Disorders of amino-acid transport |
|  | N25.1 | Nephrogenic diabetes insipidus |
|  | N25.8 | Other disorders resulting from impaired renal tubular function |
| Obstructive nephropathy | N13.0 | Hydronephrosis with obstruction of the ureteropelvic junction |
|  | N13.1 | Hydronephrosis with obstruction of the ureteropelvic junction |
|  | N13.2 | Hydronephrosis with renal and ureteral-calculous obstruction |
|  | N13.6 | Pyonephrosis |
|  | N13.8 | Other obstructive and reflux uropathy |
| Congenital and hereditary nephropathy | Q60 | Renal agenesis and other reduction defects of the kidney |
|  | Q61 | Cystic kidney disease |
|  | Q63.1 | Lobulated, fused and horseshoe kidney |
|  | Q63.2 | Ectopic kidney |
|  | Q63.9 | Congenital malformation of kidney, unspecified |
|  | P96.0 | Congenital renal failure |
|  | Q87.801 | Alport syndrome |
|  | N07 | Hereditary nephropathy, not elsewhere classified |
|  | Q27.1 | Congenital renal artery stenosis |
| Autoimmune diseases | M32.1 + N08.5 | Systemic lupus erythematosus + Glomerular disorders |
|  | M32.1 + N16.4 | Systemic lupus erythematosus + Renal  tubulointerstitial diseases |
|  | D69 | Purpuric nephritis in “Purpura and other hemorrhagic conditions” |
|  | M00–M25 | Kidney injury in “Arthropathies” |
|  | M30 | Kidney injury in “Polyarteritis nodosa and related conditions” |
|  | M31.0 + N08.5 | Goodpasture syndrome + Glomerular disorders |
|  | M31.001 | Goodpasture syndrome associated with glomerulonephritis |
|  | M31.7 + N08.5 | Microscopic polyangiitis + Glomerular disorders |
|  | M31.802 | ANCA-associated glomerulonephritis |
|  | M31.102 + N08.5 | Thrombotic thrombocytopenic purpura associated with glomerular disorders |
|  | M31.303 + N08.5 | Wegener granulomatosis associated with glomerular disorders |
|  | M31.4 + I15.0 | Aortic arch syndrome + Renovascular  hypertension |
|  | M34 | Systemic sclerosis kidney damage in “Systemic sclerosis” |
|  | M35.0 + N16.4 | Sjögren’s syndrome + Renal  tubulointerstitial diseases |
| Neoplastic diseases | C90.0 + N16.1 | Multiple myeloma + Renal tubulointerstitial disorders in neoplastic diseases |
|  | C64 | Malignant neoplasm of kidney |
|  | C79.0 | Secondary malignant neoplasm of kidney and renal pelvis |
|  | C76–C80 | Renal cancer in “Malignant neoplasm of other and ill-defined sites. Secondary and unspecified malignant neoplasm of lymph nodes. Secondary malignant neoplasm of respiratory and digestive organs. Secondary malignant neoplasm of other sites. Malignant neoplasm without specification of site” |
|  | Z51.1, Z98.8 | Renal cancer in “Chemotherapy session for neoplasm. Other specified postsurgical states” |
|  | D41.0 | Neoplasm of uncertain or unknown behavior: Kidney |
|  | E85 + N08 | Amyloidosis + Glomerular disorders in diseases classified elsewhere |
| Infection-related nephritis | B16 | HBV-related nephritis in HBV |
|  | B17 | HCV-related nephritis in HCV |
|  | B18, B19, K73 | Kidney injury in “Chronic viral hepatitis. Unspecified viral hepatitis. Chronic hepatitis, not elsewhere classified” |
|  | B20-B24 | Kidney injury in “Human immunodeficiency virus disease” |
|  | A52.7 + N08.0 | Glomerular disease in syphilis |
|  | N29.0 | Late syphilis of the kidney |
| Other related diagnoses | N26 | Unspecified contracted kidney |
|  | N28.0 | Ischemia and infarction of kidney |
|  | N28.8 | Other specified disorders of the kidney and ureter |
|  | N28.9 | Disorder of the kidney and ureter, unspecified (excluding N28.903 and N28.904) |
|  | N08.4 | Glomerular disorders in other endocrine, nutritional and metabolic diseases |
|  | I70.1 | Atherosclerosis of renal artery |
|  | K76.7 | CKD in “Hepatorenal syndrome” |

Note: CKD, chronic kidney disease; AKI, acute kidney injury; HBV, hepatitis B; HCV, hepatitis C

Supplemental Table 3. Coding of the comorbidities of chronic kidney disease

| Comorbidities of CKD | ICD-10 code |  |
| --- | --- | --- |
| Hypertension | I10 | Essential (primary) hypertension |
|  | I11 | Hypertensive heart disease |
|  | I12 | Hypertensive renal disease |
|  | I13 | Hypertensive heart and renal disease |
|  | I15 | Secondary hypertension |
| Diabetes mellitus | E10 | Insulin-dependent diabetes mellitus |
|  | E11 | Non-insulin-dependent diabetes mellitus |
|  | E12 | Malnutrition-related diabetes mellitus |
|  | E13 | Other specified diabetes mellitus |
|  | E14 | Unspecified diabetes mellitus |
| Cardiovascular and cerebrovascular diseases |  |  |
| Acute coronary syndrome | I21 | Other forms of heart disease |
|  | I20.0 | Unstable angina |
|  | I20.1 | Angina pectoris with documented spasm |
|  | I20.9 | Angina pectoris, unspecified |
|  | I22 | Subsequent myocardial infarction |
|  | I23 | Certain current complications following acute myocardial infarction |
|  | I24 | Other acute ischemic heart diseases |
| Heart failure | I50 | Heart failure |
| Other heart diseases | I25 | Chronic ischemic heart disease |
|  | I20.8 | Other forms of angina pectoris |
|  | I26–I28 | Pulmonary heart disease and diseases of the pulmonary circulation |
|  | I05–I09 | Chronic rheumatic heart diseases |
|  | I10–I15 | Hypertensive diseases |
|  | I30–I52 | Other forms of heart disease |
| Cerebrovascular diseases | I60 | Subarachnoid hemorrhage |
|  | I61 | Intracerebral hemorrhage |
|  | I62 | Other nontraumatic intracranial hemorrhage |
|  | I63 | Cerebral infarction |
|  | I64 | Stroke, not specified as hemorrhage or infarction |
|  | I65 | Occlusion and stenosis of precerebral arteries, not resulting in cerebral infarction |
|  | I67 | Other cerebrovascular diseases |
|  | I68 | Cerebrovascular disorders in diseases classified elsewhere |
|  | I69 | Sequelae of cerebrovascular disease |
|  | G45 | Transient cerebral ischemic attacks and related syndromes |
|  | H34.1 | Occlusion of a central retinal artery |
| Infectious diseases | J12–J18 | Pneumonia |
|  | J20–J22 | Other acute infections of the lower respiratory |
|  | A00–A09 | Intestinal infectious diseases |
|  | A40 A41,  A02.1, A22.7, A26.7 | Septicemia |
|  | A50–A53, A65 | Syphilis |
|  | A15–A19 | Tuberculosis |
|  | B16 | Acute infection with the hepatitis B virus |
|  | B17.0 | Acute delta-(super)infection of hepatitis B carrier |
|  | B18.0 | Chronic infection with the hepatitis B virus with a delta-agent |
|  | B18.1 | Chronic infection with the hepatitis B virus without a delta-agent |
|  | B17.1 | Acute infection with the hepatitis C virus |
|  | B18.2 | Chronic infection with the hepatitis C virus |
|  | B15–B19 | Viral hepatitis (excluding Hepatitis B and Hepatitis) |
|  | B20–B24 | Human immunodeficiency virus disease |
| Neoplasms | C00–C97 | Malignant neoplasms (excluding a malignant neoplasm of the kidney) |
|  | D00–D09 | *In situ* neoplasms |
|  | D10–D36 | Benign neoplasms |
|  | D37–D48 | Neoplasms of uncertain or unknown behavior (excluding D41.0) |
| Liver diseases | K70–K77 | Liver diseases (excluding HBV, HCV) |
| Systemic connective-tissue disorders | M30–M36 | Systemic connective-tissue disorders |
|  | M05 | Seropositive rheumatoid arthritis |
|  | M06 | Other rheumatoid arthritis |
|  | I77.6 | Arteritis, unspecified |
| Diseases of the digestive system | K25-K28 | Digestive ulcer |
|  | K20 | Esophagitis |
|  | K21 | Gastro-esophageal reflux disease |
|  | K22, K23 | Other diseases of the esophagus |
|  | K29 | Gastritis and duodenitis |
|  | K30 | Dyspepsia |
|  | K31 | Other diseases of the stomach and duodenum |
|  | K50–K52 | Noninfective enteritis and colitis |
| Diseases of the respiratory system | J40–J42 | Bronchitis |
|  | J43 | Emphysema |
|  | J44 | Other chronic obstructive pulmonary disease |
|  | J45 | Asthma |
|  | J46 | Status asthmaticus |
|  | J47 | Bronchiectasis |
| AKI | K76.7 | Hepatorenal syndrome |
|  | T79.5 | Traumatic anuria |
|  | D59.3 | Hemolytic-uremic syndrome |
|  | N01 | Rapidly progressive nephritic syndrome |
|  | O90.4 | Postpartum acute renal failure |
|  | N99.0 | Postprocedural disorders of the genitourinary tract |
|  | N10 | Acute tubulointerstitial nephritis |
|  | A98.5 | Hemorrhagic fever with renal syndrome |
|  | N13 | AKI in “Obstructive and reflux uropathy” |
|  | N12 | AKI in “Tubulointerstitial nephritis, not specified as acute or chronic” |
|  | N14 | AKI in “Drug- and heavy metal-induced tubulointerstitial and tubular conditions” |
|  | N17 | Acute renal failure |
|  | N28.9 | AKI in “Disorder of kidney and ureter, unspecified” |
| PVD | I70-I79 | Diseases of arteries, arterioles, and capillaries |
|  | I80-I89 | Diseases of veins, lymphatic vessels, and lymph nodes, not elsewhere classified |
| Dementia | G30，F00 | Alzheimer's disease, Dementia in Alzheimer’s disease |
|  | F01 | Vascular dementia |
|  | F02, F03 | Other dementia |

Note: CKD, chronic kidney disease; HBV, hepatitis B; HCV, hepatitis C; AKI, acute kidney injury; PVD, peripheral vascular disease

Supplemental Table 4. Patients with chronic kidney disease stratified by age and sex

| Age (years) | Gender | | | | Total | |
| --- | --- | --- | --- | --- | --- | --- |
|  | Male | | Female | |  |  |
|  | Total | CKD  (n, %) | Total | CKD (n, %) | Total | CKD  (n, %) |
| ≥65 | 85,745 | 3982 (4.6) | 68485 | 2984 (4.4) | 154231 | 6966 (4.5) |
| 18–64 | 13,6045 | 11,468 (8.4) | 90182 | 7392 (8.2) | 226,230 | 18,860 (8.3) |
| 18–44 | 32112 | 5375 (16.7) | 16462 | 2723 (16.5) | 48575 | 8098 (16.7) |
| 45–64 | 103933 | 6093 (5.9) | 73720 | 4669 (6.3) | 17,7655 | 10,762 (6.1) |
| Total | 221,790 | 15,450 (7.0) | 158,667 | 10,376 (6.5) | 38,0461 | 25,826 (6.8) |

Note: CKD, chronic kidney disease
